# Rebreathing‐induced hypoxemia improves glucose tolerance in adults with type 2 diabetes

**DOI:** 10.14814/phy2.70596

**Published:** 2025-10-15

**Authors:** Jiahui Zhao, Jamie G. Guei, Sophie Lalande

**Affiliations:** ^1^ Department of Kinesiology and Health Education The University of Texas at Austin Austin Texas USA

**Keywords:** glucose, hypoxia, oxygen saturation, type 2 diabetes

## Abstract

Hypoxia stimulates glucose uptake in isolated skeletal muscle through an insulin‐independent pathway. Intermittent hypoxia can lower glucose concentration in adults with type 2 diabetes, but its application remains limited by the use of gas tanks to induce hypoxia. The aim of this study was to examine the effect of rebreathing‐induced hypoxia on glucose and insulin responses to an oral glucose tolerance test in adults with type 2 diabetes. Ten adults with type 2 diabetes performed an oral glucose tolerance test during either rebreathing‐induced hypoxia or spontaneous breathing. The glucose and insulin responses to the oral glucose tolerance test did not differ between rebreathing‐induced hypoxia and spontaneous breathing. However, participants who achieved hypoxemia, defined as an oxygen saturation nadir below 90%, during rebreathing‐induced hypoxia (*n* = 5) showed lower glucose concentrations and glucose area under the curve (AUC) (20,376 ± 553 vs. 24,346 ± 639, *p* < 0.01) than participants who achieved an oxygen saturation nadir above 90% (*n* = 5). Interestingly, body weight was strongly correlated with oxygen desaturation (*r* = −0.87, *p* < 0.01) and glucose AUC (*r* = −0.81, *p* < 0.01) during rebreathing‐induced hypoxia. Rebreathing‐induced hypoxia may represent a promising strategy to improve glycemic control in adults with type 2 diabetes and coexisting obesity.

## 1 INTRODUCTION

Type 2 diabetes increases the risk of macrovascular and microvascular complications and leads to a greater risk of premature death in more than 38 million U.S. adults (Baena‐Diez et al., 2016; Centers for Disease Control and Prevention, 2024). Stringent glycemic control, defined as glycated hemoglobin (HbA1c) values below 7%, lowers the risk of diabetes‐related illness and death (Boye et al., 2022). Despite a similar use of glucose‐lowering medication, glycemic control recently declined among adults with diabetes (Fang et al., 2021). Indeed, the percentage of adults with diabetes achieving glycemic control fell from 57.4% in 2007–2010 to 50.5% in 2015–2018 (Fang et al., 2021), and 47.4% of U.S. adults diagnosed with diabetes had an HbA1c value above or greater than 7% in 2017–2020 (Centers for Disease Control and Prevention, 2024). Importantly, reducing HbA1c values from 9.9 to 6.8% is associated with a 3.9 years gain in life expectancy (Kianmehr et al., 2022). Postprandial glucose concentration measured 1 h after a meal represents the best predictor of HbA1c values in adults with well‐controlled type 2 diabetes (Bell, 2001; Turner et al., 1999). It is therefore imperative to develop interventions that attenuate postprandial increases in glucose concentration to improve glycemic control in adults with type 2 diabetes.

Hypoxia, or low oxygen availability, stimulates the translocation of GLUT4 transporters via the 5′ adenosine monophosphate‐activated protein kinase (AMPK) pathway, which leads to glucose uptake in isolated skeletal muscle (Hayashi et al., 2000). Importantly, this hypoxia‐activated signaling pathway acts independently of insulin‐stimulated glucose transport, which is impaired in type 2 diabetes (Azevedo Jr. et al., 1995; Cartee et al., 1991; Mu et al., 2001). In the laboratory, hypoxia is achieved by breathing air with a lower fraction of oxygen from gas tanks. Exposure to hypoxia of mild and moderate severity has a beneficial effect on glycemic control in adults with type 2 diabetes. Indeed, a single session of intermittent hypoxia, consisting of five 6‐min bouts breathing at a 13% fraction of inspired oxygen, reduced glucose concentration in adults with type 2 diabetes (Duennwald et al., 2013). Sixty minutes of continuous hypoxia at a fraction of inspired oxygen of 14.6% resulted in an improved insulin sensitivity response to a 4‐h labeled intravenous glucose tolerance test in adults with type 2 diabetes (Mackenzie et al., 2011). In addition, we demonstrated that intermittent hypoxia, consisting of eight 4‐min hypoxic cycles at a targeted arterial oxygen saturation of 80% equivalent to a fraction of inspired oxygen of 11%, improved insulin sensitivity during an oral glucose tolerance test in adults with type 2 diabetes (Zhao et al., 2025).

Despite these promising results, intermittent hypoxia requires the use of gas tanks to create a hypoxic environment and is therefore not applicable to daily life. Nonetheless, an intervention that can induce brief hypoxic conditions through a reduced fraction of inspired oxygen could attenuate the postprandial increase in glucose concentration. Fraction of inspired oxygen can also be reduced by rebreathing into a low‐volume closed‐circuit system containing ambient air. Thus, the aim of this study was to determine the effect of few bouts of rebreathing‐induced hypoxia on glucose and insulin concentrations during an oral glucose tolerance test in adults with type 2 diabetes.

## 2 MATERIALS AND METHODS

**FIGURE 1:**
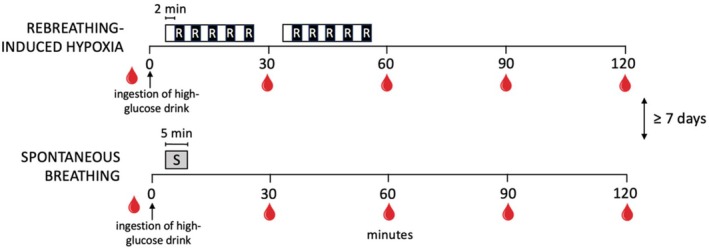
Study protocol. R, rebreathing; S, spontaneous.

This study was a randomized crossover trial. Men and women living with type 2 diabetes visited the laboratory on two occasions separated by at least 1 week. On both visits, an oral glucose tolerance test was conducted while simultaneously performing rebreathing‐induced hypoxia or while simultaneously performing spontaneous breathing (Figure 1). Participants were excluded from the study if they had uncontrolled stage 2 hypertension or were taking more than one antihypertensive medication, were current smokers, had a history of cardiovascular diseases or lung disease, or had previously been diagnosed with diabetic complications such as nephropathy, neuropathy, or retinopathy. Participants were asked to avoid intense physical activity on the day prior to both visits and reported to the laboratory after an overnight fast of at least 8 h. All individuals provided informed written consent for participating in the study, which was approved by the Institutional Review Board of the University of Texas at Austin (IRB study number 00001555).

### 2.1 Oral glucose tolerance test

A 2‐h oral glucose tolerance test was performed, with venous blood draws collected before and 30, 60, 90, and 120 min after ingestion of a 75 g glucose drink (Trutol, Thermo Scientific, MA, USA) for measurements of glucose and insulin concentrations. Blood samples were centrifuged and plasma was aliquoted and frozen at −80°C for subsequent batch analyses. Glucose concentration was determined using a single cuvette glucose assay run in duplicates using a hexokinase reagent (23‐666‐280, Pointe Scientific, MI, USA). Insulin concentration was determined using an enzyme‐linked immunosorbent assay (80‐INSHU‐E01.1, ALPCO, NH, USA). The average coefficient of variation for the glucose and insulin assays was 1.4% and 3.6%, respectively. Glucose and insulin area under the curve (AUC) were calculated using the linear trapezoidal rule formula. Insulin sensitivity was estimated with the Matsuda index using glucose and insulin concentrations obtained during the oral glucose tolerance test (Matsuda & DeFronzo, 1999). The homeostatic model assessment for insulin resistance (HOMA‐IR) was calculated by multiplying fasting insulin by fasting glucose and dividing by 22.5 (Matthews et al., 1985). HbA1c was determined from the venous blood draw obtained before ingestion of the high‐glucose drink on the first visit to the laboratory (DCA Vantage Analyzer, Siemens Healthcare GmbH, Germany).

### 2.2 Rebreathing‐induced hypoxia

The rebreathing‐induced hypoxia protocol consisted of two series of five 2‐min rebreathing bouts interspersed with 2 min of breathing room air (Figure 1). Participants were sitting during the protocol. The first series of rebreathing bouts was performed from min 5 to 25 of the oral glucose tolerance test, and the second series of rebreathing bouts was performed from min 35 to 55 of the oral glucose tolerance test (Figure 1). To induce hypoxia, participants rebreathed room air from a low‐volume closed‐circuit system consisting of a 3‐L anesthesia bag (Rusch, USA) connected to a 4‐way directional control valve (Hans Rudolph, Inc., KS, USA) with one of the ports equipped with a mouthpiece. The 3‐L anesthesia bag was fitted with a stopcock. For each rebreathing bout, the anesthesia bag was filled with air using a 1000 mL syringe. Once the rebreathing bout was completed, the bag was emptied of any remaining gases before being filled with air for the subsequent rebreathing bout. Because oxygen is not added to the closed‐circuit system during rebreathing, the fraction of oxygen in the circuit decreases with every breath during the 2‐min rebreathing bout. Rebreathing for 2 min represents the near‐maximal duration that participants can tolerate without disconnecting from the mouthpiece due to a hypercapnia‐induced respiratory drive. Ten rebreathing bouts were performed to correspond with the total duration of approximately 1 h used in previous intermittent hypoxia protocols (Duennwald et al., 2013; Zhao et al., 2025).

### 2.3 Hemodynamics, oxygen saturation and ventilation

Blood pressure and heart rate were measured after 5 min of supine rest at the beginning of each visit (Omron Healthcare, Inc., IL, USA). Hemodynamics, oxygen saturation, and ventilation were assessed throughout the rebreathing‐induced hypoxia protocol and for a duration of 5 min during spontaneous breathing (min 5–10 of the oral glucose tolerance test). Breath‐by‐breath ventilation was assessed using a metabolic cart calibrated with standardized gas and room air (Ultima Cardio2, MGC Diagnostics, MN, USA) throughout rebreathing‐induced hypoxia and spontaneous breathing. The pneumotachometer was mounted between the mouthpiece and the four‐way directional control valve of the rebreathing circuit. Brachial arterial waveform and oxygen saturation were continuously recorded during rebreathing‐induced hypoxia and spontaneous breathing using finger plethysmography and pulse oximetry, respectively (NOVA, Finapres Medical Systems, Amsterdam, The Netherlands). Blood pressure, heart rate, stroke volume, cardiac output, and total peripheral resistance were derived from the brachial arterial waveform using the Modelflow® method (Wesseling et al., 1985). All data were recorded using LabChart software for later analyses (Powerlab, ADInstruments Inc., CO, USA).

### 2.4 Data and statistical analysis

Breath‐by‐breath ventilation, beat‐by‐beat hemodynamics, and oxygen saturation were averaged every 10 s throughout rebreathing‐induced hypoxia and spontaneous breathing. The oxygen saturation nadir was identified for each rebreathing bout, and an average oxygen saturation nadir was calculated for the 10 rebreathing bouts. Average 1‐min values for hemodynamics and ventilatory variables were calculated throughout the rebreathing‐induced hypoxia protocol. For each variable, the last minute of each rebreathing bout was averaged over the 10 rebreathing bouts. Average values for hemodynamics and ventilatory variables were calculated over the 5 min of spontaneous breathing. A two‐way repeated measures analysis of variance was used to evaluate the effect of condition (rebreathing‐induced hypoxia vs. spontaneous breathing) and time (min 0, 30, 60, 90, and 120) on glucose and insulin concentrations. When appropriate, post‐hoc analyses were performed using Tukey's test. A paired t‐test was used to test the effect of condition (rebreathing‐induced hypoxia vs. spontaneous breathing) on insulin sensitivity, glucose AUC, insulin AUC, ventilatory, and hemodynamics variables. Pearson's correlation was used to determine the relation between variables. The previously observed difference in peak glucose concentration during an oral glucose tolerance test conducted under intermittent hypoxia versus a sham protocol in adults with type 2 diabetes was used to calculate sample size (Zhao et al., 2025). With a power of 0.80 and an alpha of 0.05, a minimum sample size of five participants was needed to detect the effect of rebreathing‐induced hypoxia on glucose tolerance. Data were further analyzed by separating participants according to their average oxygen saturation nadir during rebreathing‐induced hypoxia. A three‐way analysis of variance with repeated measures was used to evaluate the effect of condition (rebreathing‐induced hypoxia vs. spontaneous breathing), group (oxygen saturation nadir above 90% vs. oxygen saturation nadir below 90%), and time (minutes 0, 30, 60, 90, and 120) on glucose and insulin concentrations. *p* < 0.05 was considered statistically significant. All values are reported as means ± standard deviations unless noted otherwise.

## 3 RESULTS

### 3.1 Participants

Ten adults with type 2 diabetes (4 women, age: 53 ± 10 years, height: 174 ± 9 cm, body weight: 101.4 ± 16.9 kg, body mass index: 34.1 ± 7.5 kg/m^2^, systolic blood pressure: 129 ± 8 mmHg, diastolic blood pressure: 83 ± 8 mmHg, heart rate: 78 ± 9 bpm) participated in the study. Participants were White (*n* = 5), Asian (*n* = 1), Latino/Hispanic (*n* = 3), and Black (*n* = 1). Diabetes duration averaged 9 ± 6 years, and HbA1c was 7.4 ± 1.0%. Participants were treated with metformin (*n* = 7), glucagon‐like peptide‐1 receptor agonists (*n* = 6), thiazolidinediones (*n* = 2), insulin glargine (*n* = 1), angiotensin‐converting enzyme inhibitors (*n* = 2), calcium channel blocker (*n* = 1), angiotensin II receptor blocker (*n* = 1), diuretic (*n* = 1), statins (*n* = 5), levothyroxine (*n* = 2), and fibrates (*n* = 1). Self‐reported physical activity levels were 2.2 ± 2.3 hours/week. All women were postmenopausal. Both visits were separated by an average of 17 ± 20 days.

### 3.2 Hemodynamics and ventilatory responses

**FIGURE 2:**
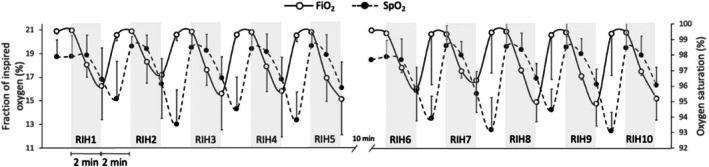
One‐minute averages of the fraction of inspired oxygen (FiO_2_) and oxygen saturation (SpO_2_) responses to 10 cycles of rebreathing‐induced hypoxia (RIH) in adults with type 2 diabetes (*n* = 10, 4 women). Note the delayed oxygen saturation response reflecting circulation time from the lungs to the periphery, and that this figure depicts 1‐min averages and not the oxygen saturation nadirs.

The average fraction of inspired oxygen and oxygen saturation responses to rebreathing‐induced hypoxia are presented in Figure 2. By design, oxygen saturation was lower during rebreathing‐induced hypoxia than during spontaneous breathing (Table 1). Heart rate, systolic blood pressure, and mean arterial pressure were higher during rebreathing‐induced hypoxia, whereas diastolic blood pressure, stroke volume, cardiac output, and total peripheral resistance remained unchanged in comparison with spontaneous breathing (Table 1). Fraction of inspired oxygen and end‐tidal oxygen were lower, respiratory rate remained unchanged, and tidal volume, minute ventilation, and end‐tidal carbon dioxide were greater during rebreathing‐induced hypoxia in comparison with spontaneous breathing (Table 1).

**TABLE 1:** Hemodynamics and ventilatory responses during rebreathing‐induced hypoxia and spontaneous breathing.

Variables	Spontaneous	Hypoxia	*p* value
Oxygen saturation, %	98 ± 1	89 ± 4	<0.01
Systolic blood pressure, mmHg	127 ± 8	142 ± 10	<0.01
Diastolic blood pressure, mmHg	83 ± 7	87 ± 8	0.07
Mean arterial pressure, mmHg	97 ± 6	105 ± 7	0.01
Heart rate, bpm	81 ± 8	88 ± 9	<0.01
Stroke volume, mL	107 ± 21	104 ± 24	0.59
Cardiac output, L/min	8.8 ± 2.3	9.3 ± 2.7	0.36
Total peripheral resistance, mmHg/L/min	11.9 ± 3.3	12.6 ± 4.5	0.43
Respiratory rate, breaths/min	12 ± 5	12 ± 3	0.66
Tidal volume, mL	919 ± 391	2611 ± 759	<0.01
Minute ventilation, L/min	9.7 ± 1.6	25.1 ± 6.2	<0.01
End‐tidal carbon dioxide, mmHg	35.1 ± 3.7	39.8 ± 5.4	0.01
End‐tidal oxygen, mmHg	105 ± 7	83 ± 11	<0.01
Fraction of inspired oxygen, %	20.9 ± 0.1	13.6 ± 3.0	<0.01

### 3.3 Glucose and insulin responses to the oral glucose tolerance test

**FIGURE 3:**
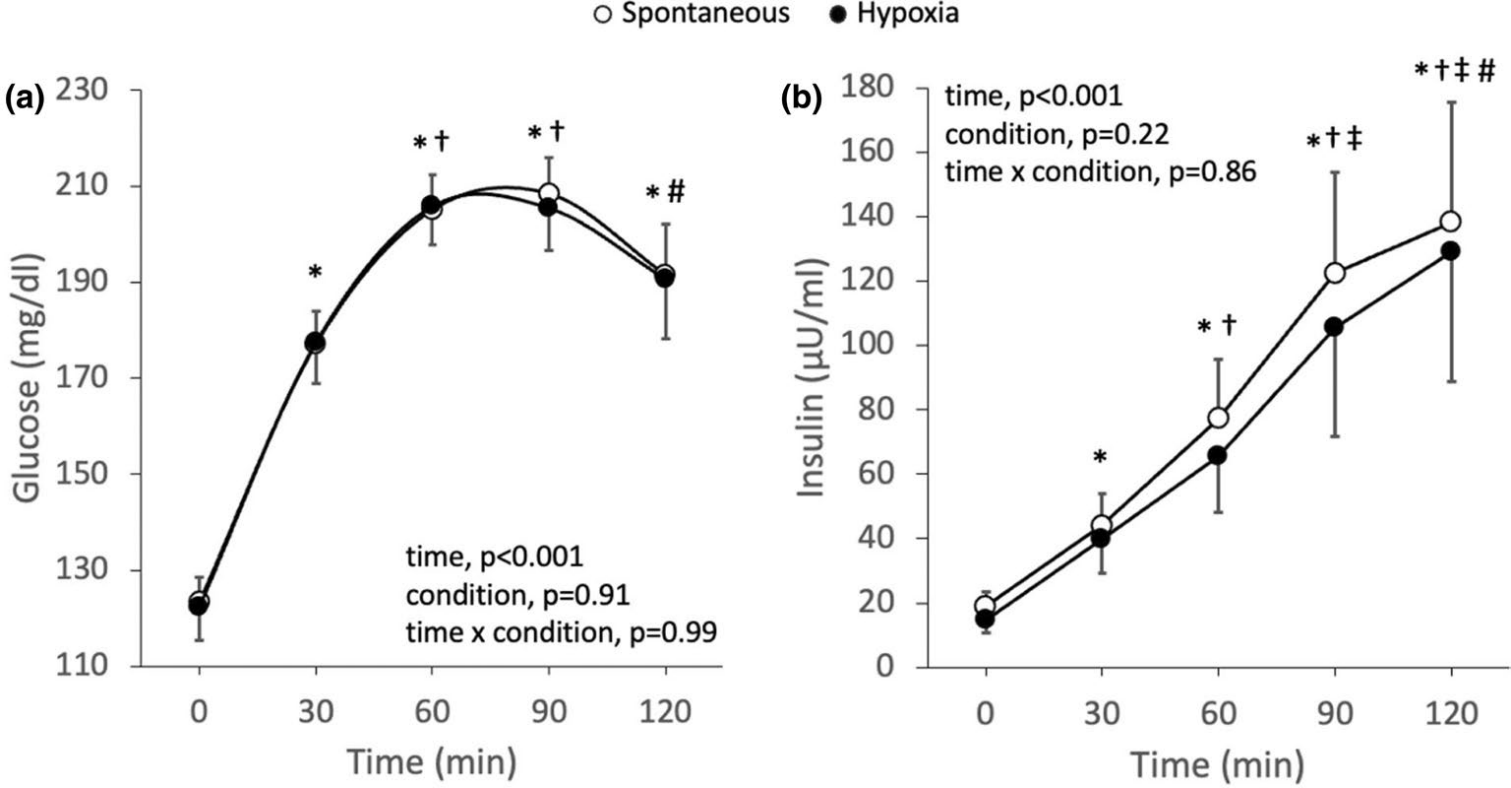
Glucose (a) and insulin (b) concentration response to an oral glucose tolerance test conducted while simultaneously performing rebreathing‐induced hypoxia or spontaneous breathing in adults with type 2 diabetes (*n* = 10, 4 women). A two‐way repeated measures analysis of variance was used to evaluate the effect of condition (rebreathing‐induced hypoxia vs. spontaneous breathing) and time (0, 30, 60, 90, and 120 min) on glucose and insulin concentrations. * Different from min 0, † different from min 30, ‡ different from min 60, # different from min 90. Values are presented as averages ± SEM.

**FIGURE 4:**
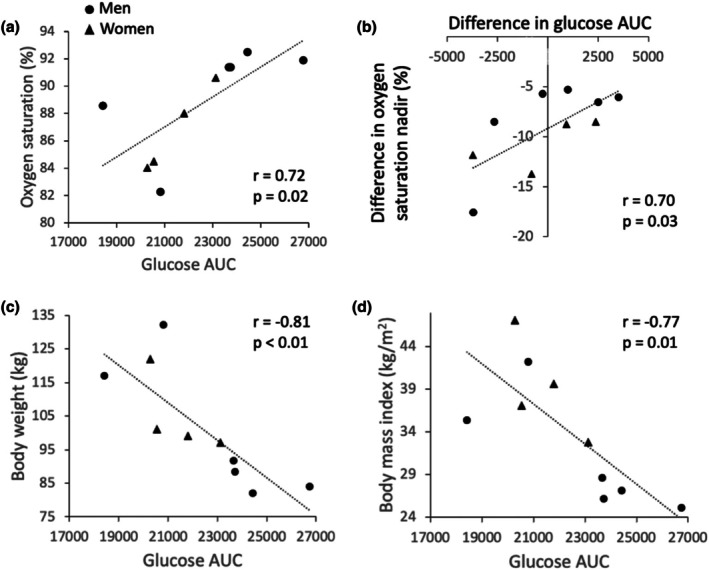
Relation between glucose AUC in response to an oral glucose tolerance test conducted while simultaneously performing rebreathing‐induced hypoxia and oxygen saturation nadir (a), body weight (c), and body mass index (d), and relation between difference in oxygen saturation nadir and difference in glucose AUC between the oral glucose tolerance test conducted while simultaneously performing rebreathing‐induced hypoxia and spontaneous breathing (b) (*n* = 10, 4 women). Pearson's correlation was used to determine the relations between these variables.

Glucose and insulin concentration increased similarly during the oral glucose tolerance test conducted while simultaneously performing rebreathing‐induced hypoxia and the oral glucose tolerance test conducted while simultaneously performing spontaneous breathing (Figure 3). Accordingly, there was no difference in insulin sensitivity (5.2 ± 6.6 vs. 4.3 ± 5.6, *p* = 0.23), insulin AUC (8485 ± 7640 vs. 9673 ± 7256, *p* = 0.18), glucose AUC (22361 ± 2443 vs. 22440 ± 1952, *p* = 0.93) or peak relative increase in glucose concentrations (99 ± 25 vs. 96 ± 19 mg/dL, *p* = 0.71) between the oral glucose tolerance test conducted while simultaneously performing rebreathing‐induced hypoxia and the oral glucose tolerance test conducted while simultaneously performing spontaneous breathing, respectively. However, there was a strong correlation between glucose AUC and the oxygen saturation nadir achieved during rebreathing‐induced hypoxia (Figure 4a), and between the difference in glucose AUC and the difference in oxygen saturation nadir between rebreathing‐induced hypoxia and spontaneous breathing (Figure 4b). Participants who experienced greater oxygen desaturation during rebreathing‐induced hypoxia showed an attenuated glucose response during the oral glucose tolerance test. Additionally, glucose AUC during rebreathing‐induced hypoxia was inversely correlated with body weight and body mass index, with heavier participants showing lower glucose AUC (Figure 4c,d). In contrast, glucose AUC during spontaneous breathing was not correlated with body weight (*r* = −0.12, *p* = 0.75) or body mass index (*r* = −0.27, *p* = 0.44). Oxygen saturation during rebreathing‐induced hypoxia was highly correlated with both body weight (*r* = −0.87, *p* < 0.01) and body mass index (*r* = −0.90, *p* < 0.01), indicating that heavier individuals experienced greater desaturation. Glucose AUC during rebreathing‐induced hypoxia was initially correlated to insulin sensitivity; however, this correlation was no longer significant after excluding an outlier data point that exceeded two standard deviations from the mean (*r* = 0.34, *p* = 0.37). There were no correlations between glucose AUC during rebreathing‐induced hypoxia and insulin AUC (*r* = −0.53, *p* = 0.11) or insulin resistance (*r* = −0.39, *p* = 0.26). Similarly, glucose AUC was not correlated with insulin AUC (*r* = 0.06, *p* = 0.86), insulin sensitivity (*r* = 0.24, *p* = 0.51) or insulin resistance (*r* = 0.01, *p* = 0.98) during spontaneous breathing.

### 3.4 Glucose and insulin responses to the oral glucose tolerance test according to oxygen saturation nadir

**FIGURE 5:**
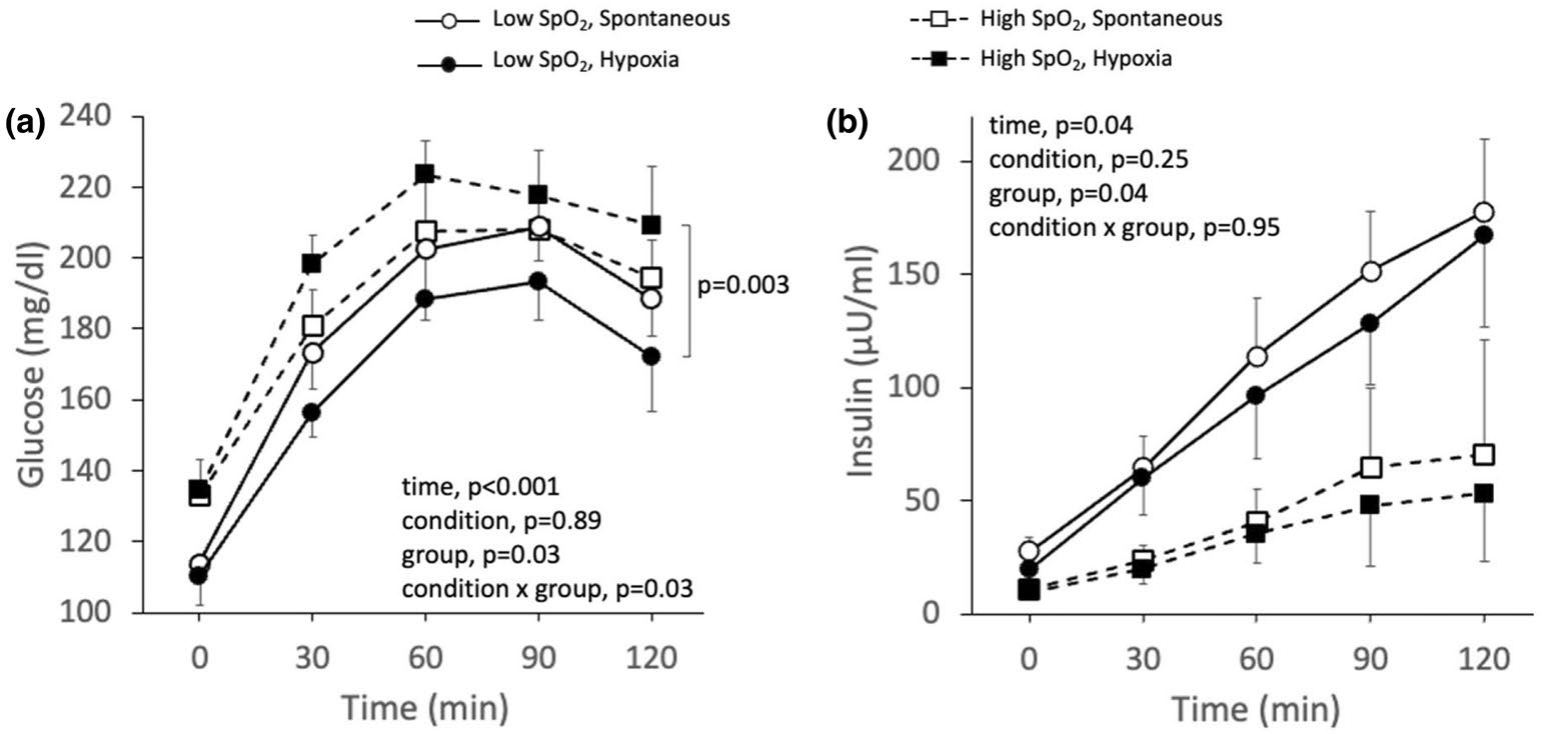
Glucose (a) and insulin (b) concentration response to an oral glucose tolerance test conducted while simultaneously performing rebreathing‐induced hypoxia or spontaneous breathing in adults with type 2 diabetes who achieved an oxygen saturation nadir below 90% (low SpO_2_, *n* = 5, 3 women) and adults with type 2 diabetes who achieved an oxygen saturation nadir above 90% (high SpO_2_, *n* = 5, 1 woman). A three‐way analysis of variance with repeated measures was used to evaluate the effect of condition (rebreathing‐induced hypoxia vs. spontaneous breathing), group (low SpO_2_ vs. high SpO_2_), and time (minutes 0, 30, 60, 90, and 120) on glucose and insulin concentrations. Values are presented as averages ± SEM.

**FIGURE 6:**
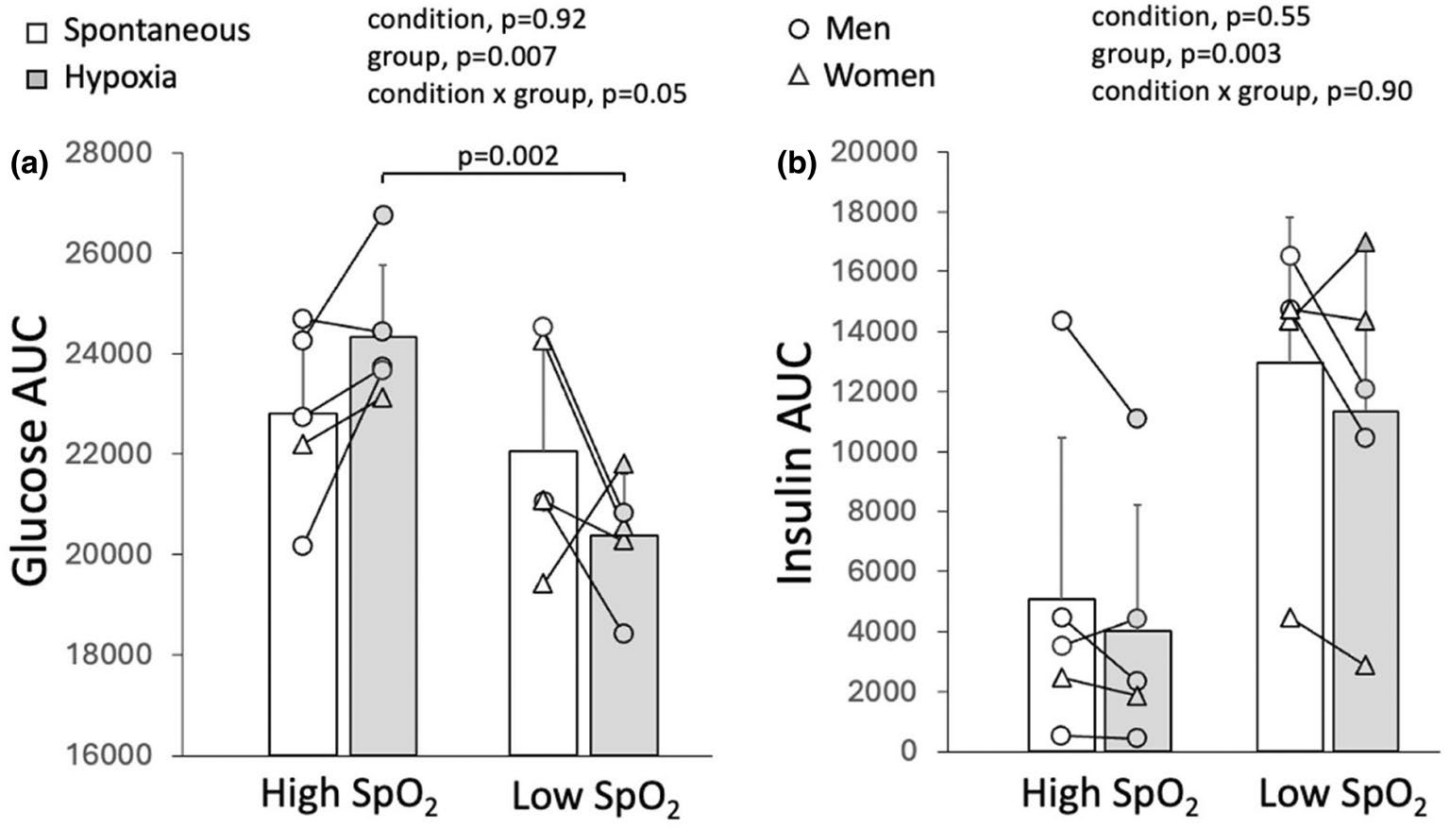
Glucose AUC (a) and insulin AUC (b) responses to an oral glucose tolerance test conducted while simultaneously performing rebreathing‐induced hypoxia or spontaneous breathing in adults with type 2 diabetes who achieved an oxygen saturation nadir above 90% (high SpO_2_, *n* = 5, 1 woman) and adults with type 2 diabetes who achieved an oxygen saturation nadir below 90% (low SpO_2_, *n* = 5, 3 women). A two‐way repeated measures analysis of variance was used to evaluate the effect of condition (rebreathing‐induced hypoxia vs. spontaneous breathing) and group (low SpO_2_ vs. high SpO_2_) on glucose and insulin AUC.

Because of the correlation between oxygen saturation nadir and the glucose AUC response to the oral glucose tolerance test, data were further analyzed by separating participants according to their average oxygen saturation nadir during rebreathing‐induced hypoxia. Hypoxemia is generally defined as an oxygen saturation below 90%; thus, participants were divided into two groups based on whether they achieved an oxygen saturation nadir above 90% (high saturation, *n* = 5) or below 90% (low saturation, *n* = 5) during rebreathing‐induced hypoxia. There was an interaction between condition and oxygen saturation nadir for glucose concentration and glucose AUC during the oral glucose tolerance test (Figures 5a and 6a). During rebreathing‐induced hypoxia, participants in the low saturation group showed lower glucose concentrations and glucose AUC in comparison with participants in the high saturation group. There was no difference in glucose concentration or glucose AUC during spontaneous breathing between the high and low saturation groups. Insulin concentration and insulin AUC were greater in participants in the low saturation group in comparison with participants in the high saturation group (Figures 5b and 6b). In accordance with the correlations observed in the whole group, participants in the low saturation group were heavier and had a greater body mass index than participants in the high saturation group. There were no differences in insulin resistance or insulin sensitivity obtained during spontaneous breathing between participants in the low and high saturation groups (Table 2). Differences in peak relative increase in glucose concentrations, hemodynamics, and ventilatory variables between rebreathing‐induced hypoxia and spontaneous breathing were similar for both groups, besides a greater decrease in oxygen saturation in the low saturation group than the high saturation group (Table 2).

**TABLE 2:** Participants' characteristics and difference in hemodynamics and ventilatory variables between rebreathing‐induced hypoxia and spontaneous breathing for the high and low saturation groups.

Variables	High saturation SpO_2_>90%	Low saturation SpO_2_<90%	*p* value
Women/Men	1/4	3/2	
Race/Ethnicity			
White	4	1	
Asian	1		
Black		1	
Latino/Hispanic		3	
Age, years	53.2 ± 5.8	53.0 ± 13.5	0.98
Height, cm	178 ± 5	169 ± 10	0.10
Body weight, kg	88.6 ± 6.0	114.2 ± 14.1	0.01
Body mass index, kg/m^2^	27.9 ± 3.0	40.3 ± 4.6	<0.01
Diabetes duration, years	11 ± 7	7 ± 5	0.29
HbA1c, %	7.6 ± 1.4	7.2 ± 0.6	0.52
HOMA‐IR	3.4 ± 2.5	7.9 ± 4.2	0.08
Insulin sensitivity	7.2 ± 7.0	1.5 ± 0.7	0.15
Δ Peak relative increase glucose, mg/dL	12 ± 14	−7 ± 27	0.19
Δ SpO_2_, %	−6 ± 2	−12 ± 4	0.02
Δ Heart rate, bpm	4 ± 3	9 ± 4	0.06
Δ Stroke volume, mL	5 ± 25	−12 ± 4	0.22
Δ Cardiac output, L/min	1.0 ± 2.1	0.0 ± 0.7	0.35
Δ Mean arterial pressure, mmHg	8 ± 5	8 ± 10	0.87
Δ Total peripheral resistance, mmHg/L/min	1.0 ± 3.9	0.5 ± 2.0	0.81
Δ Respiratory rate, breaths/min	−1 ± 3	0 ± 3	0.45
Δ Tidal volume, mL	1512 ± 766	1872 ± 609	0.44
Δ Minute ventilation, L/min	14.3 ± 7.2	16.5 ± 4.8	0.58
Δ End‐tidal CO_2_, mmHg	4.7 ± 3.7	4.7 ± 6.3	1.00
Medication use, *n*			
Metformin	2	5	
Glucagon‐like peptide‐1 receptor agonists	2	4	
Thiazolidinediones	2		
Insulin glargine		1	
Angiotensin converting enzyme inhibitor			
Calcium channel blocker	1		
Angiotensin II receptor blocker	1	2	
Diuretic	1		
Statins	3	2	
Levothyroxine		2	
Fibrates	1		

Abbreviation: SpO_2_, oxygen saturation.

## 4 DISCUSSION

The present study sought to determine the effect of a few bouts of rebreathing‐induced hypoxia on the glucose and insulin responses to an oral glucose tolerance test in adults with type 2 diabetes. There was no difference between the glucose and insulin responses to the oral glucose tolerance test conducted while simultaneously performing rebreathing‐induced hypoxia and the oral glucose tolerance test conducted while simultaneously performing spontaneous breathing across all participants. However, there was a strong correlation between the achieved oxygen saturation nadir during rebreathing‐induced hypoxia and the glucose response to the oral glucose tolerance test. Participants who achieved an oxygen saturation nadir below 90% during rebreathing‐induced hypoxia showed lower glucose concentrations and glucose AUC than participants who achieved an oxygen saturation nadir above 90%, indicating an improved glucose tolerance in participants who experienced hypoxemia.

Intermittent hypoxia, administered using gas tanks, has shown equivocal findings regarding glucose regulation in adults with type 2 diabetes (Duennwald et al., 2013; Zhao et al., 2025). Intermittent hypoxia, consisting of five 6‐min bouts breathing at a 13% fraction of inspired oxygen, immediately reduced glucose concentration in adults with type 2 diabetes. However, the subsequent ingestion of a meal resulted in similar 3‐h postprandial increases in glucose concentration between intermittent hypoxia and a placebo condition consisting of breathing room air (Duennwald et al., 2013). Similarly, intermittent hypoxia, consisting of eight 4‐min cycles at an oxygen saturation of 81% equivalent to a fraction of inspired oxygen of 11%, did not attenuate the increase in glucose concentration during an oral glucose tolerance test in adults with type 2 diabetes (Zhao et al., 2025). Continuous hypoxia at a fraction of inspired oxygen of 14.6% and intermittent hypoxia, consisting of eight 4‐min hypoxic cycles at a targeted arterial oxygen saturation of 80%, improved insulin sensitivity in response to a 4‐h labeled intravenous glucose tolerance test and during an oral glucose tolerance test in adults with type 2 diabetes, respectively (Mackenzie et al., 2011; Zhao et al., 2025). Contrary to these previous hypoxia protocols using gas tanks to control the fraction of inspired oxygen or oxygen saturation, rebreathing‐induced hypoxia did not target a specific fraction of inspired oxygen or oxygen saturation. Instead, participants performed rebreathing, which resulted in varying levels of oxygen desaturation. Notably, participants who achieved hypoxemia during rebreathing‐induced hypoxia showed lower glucose concentrations and glucose AUC in response to the oral glucose tolerance test than participants who did not achieve hypoxemia. Moreover, the strong correlation between the difference in oxygen saturation and the difference in glucose AUC between rebreathing‐induced hypoxia and spontaneous breathing supports the role of hypoxemia in driving improvements in glucose tolerance. Thus, greater hypoxic severity during rebreathing‐induced hypoxia may be necessary to induce improvements in glucose tolerance in adults with type 2 diabetes. It is noteworthy that intermittent hypoxia at an oxygen saturation nadir of 81% did not improve glucose tolerance (Zhao et al., 2025), suggesting that hypoxemia alone is not the sole factor contributing to the improved glucose tolerance observed during rebreathing‐induced hypoxia. Unlike intermittent hypoxia, rebreathing‐induced hypoxia triggered an increase in mean arterial pressure, which may have enhanced skeletal muscle perfusion and thereby facilitated glucose delivery. It is therefore possible that the greater hemodynamic challenge associated with rebreathing‐induced hypoxia contributes to the observed improved glucose tolerance in adults with type 2 diabetes.

Body weight and body mass index were not correlated with the glucose response to the oral glucose tolerance test during spontaneous breathing. However, both body weight and body mass index were strongly correlated with oxygen saturation and the glucose response during rebreathing‐induced hypoxia. Participants in the low saturation group were, on average, 25 kg heavier and had a higher body mass index than participants in the high saturation group. The greater oxygen desaturation observed in heavier participants may be due to excess body weight, as obesity profoundly affects pulmonary gas exchange. Indeed, even in the absence of changes in lung volumes, individuals living with obesity display low ventilation‐perfusion ratios (Rivas et al., 2015; Rochester & Enson, 1974), which likely contribute to hypoxemia under hypoxic conditions. Accordingly, body mass index represents an important predictor of the severity of oxygen desaturation during apnea (Peppard et al., 2009). It is therefore possible that abnormal ventilation‐perfusion distribution contributed to the greater desaturation observed during rebreathing‐induced hypoxia in individuals with obesity.

Insulin concentration and insulin AUC responses to the oral glucose tolerance tests were greater in the low saturation group in comparison with the high saturation group. These findings are consistent with the greater, although not statistically significant, insulin resistance observed in the low saturation group. Indeed, insulin resistance leads to compensatory hyperinsulinemia as pancreatic insulin secretion increases to maintain glucose homeostasis (Kahn et al., 1993). It is therefore possible that insulin resistance contributed to the improved glucose tolerance observed in the low saturation group. However, it is important to note that the divergent glucose responses to the oral glucose tolerance test observed between the low and high saturation groups during rebreathing‐induced hypoxia were not accompanied by corresponding differences in insulin responses. The improved glucose tolerance observed in the low saturation group may reflect hypoxia‐induced activation of the alternative AMPK pathway as a result of impaired insulin signaling.

### 4.1 Limitations

While the study was adequately powered to address the primary research objective, the sample size for the subgroup analysis was small, limiting the generalizability of these findings. Additionally, the present study design did not target a specific level of oxygen desaturation during rebreathing‐induced hypoxia. Based on the present findings, future studies should aim to achieve an oxygen saturation below 90% during rebreathing, either by reducing the volume of air in the bag or by increasing the respiratory rate, in a larger sample of adults with type 2 diabetes. There were no inclusion criteria restricting the type of medication used to treat type 2 diabetes. Although the timing of the last medication dose was not assessed, participants acted as their own control in this randomized crossover trial, and medication use remained consistent throughout the short time course of the study. However, it is possible that medication use and/or timing influenced the glucose and insulin responses to the oral glucose tolerance tests. Finally, activation of the AMPK pathway in response to rebreathing‐induced hypoxia was not confirmed by muscle biopsies. Therefore, it can only be speculated that hypoxemia stimulates glucose uptake in humans via the same mechanisms identified in isolated skeletal muscle preparations.

In conclusion, rebreathing‐induced hypoxia did not affect the glucose and insulin responses to an oral glucose tolerance test in the group as a whole. However, rebreathing‐induced hypoxemia improved glucose tolerance in adults with type 2 diabetes. These results suggest that rebreathing‐induced hypoxia should cause hypoxemia, as defined by an oxygen saturation below 90%, to induce an improved glucose tolerance in adults with type 2 diabetes. Despite the use of glucose‐lowering medication, nutrition, and physical activity to treat type 2 diabetes, almost half of U.S. adults diagnosed with diabetes do not achieve the recommended HbA1c of less than 7% (American Diabetes Association, 2021; American Diabetes Association, 2025; Centers for Disease Control and Prevention, 2024). Modulation of postprandial glycemia plays a particularly important role in overall glycemic control (Bell, 2001), therefore, postprandial glycemia represents a crucial target for intervention to prevent complications associated with type 2 diabetes (American Diabetes Association, 2001). Rebreathing‐induced hypoxia may represent a novel, accessible, and affordable strategy to acutely reduce postprandial hyperglycemia in adults with type 2 diabetes. Future studies should confirm its effectiveness in improving glucose tolerance and establish its safety, tolerability, and feasibility, especially in clinical populations at risk of ventilatory impairments, such as individuals with obesity.

## AUTHOR CONTRIBUTIONS

Sophie Lalande conceived and designed research. Jiahui Zhao and Jamie G. Guei performed experiments. Jiahui Zhao analyzed data; Jiahui Zhao and Sophie Lalande interpreted results of experiments. Sophie Lalande prepared figures; Jiahui Zhao and Sophie Lalande drafted manuscript. Jamie G. Guei edited and revised manuscript. All authors approved the final version of the manuscript.

## FUNDING INFORMATION

This work was supported by the Small Grants Program from the College of Education of the University of Texas at Austin (Sophie Lalande).

## CONFLICT OF INTEREST STATEMENT

No conflicts of interest, financial or otherwise, are declared by the authors.

## Data Availability

The datasets generated and/or analyzed during the current study are available from the corresponding author upon reasonable request.
